# Triumphant Massachusetts

**Published:** 1896-07

**Authors:** 


### TRIUMPHANT MASSACHUSETTS.

THE sober second thought of an intelligent and thinking people generally proves sound and trustworthy, and the outcome of the thorough open discussion of the whole subject of tuberculosis and its every aspect and phase from the point of view of the Tuberculosis Commission of the Bay State has won for the people of that Commonwealth the most significant and far-reaching victory achieved in the past ten years. The appropriation asked for the present year to continue the good work (being done there for the whole world) was raised fifty thousand dollars above the original appropriation asked for, and the commission will have at its command three hundred thousand dollars, which we are fully sure will bring more than that measure of benefit to the people of Massachusetts, not to mention the great benefits indirectly derived by the progressive people the world over. This tribute to the worth of a commission whose zeal and efforts have been of the most earnest, sincere, and intelligent character must come to them as a solace and comfort, after the smoke and fury of the battle have passed away. While it places a greater responsibility than ever upon their shoulders, none who are acquainted with the high character of the men comprising the commission will not for a moment have the slightest fear or anxiety that these higher and graver duties will be discharged with the same fidelity and faithfulness as in the past. All honor to the legislative servants of the people of Massachusetts! All praise to the confidence reposed in her commission, and well done to these good and faithful servants, who, while working within the confines of a State, are nevertheless doing the world's work!

A lesson for every commonwealth to study.

